# Transparent Interaction Based Learning for Human-Robot Collaboration

**DOI:** 10.3389/frobt.2022.754955

**Published:** 2022-03-04

**Authors:** Elahe Bagheri, Joris De Winter, Bram Vanderborght

**Affiliations:** Robotics and Multibody Mechanics Research Group, Vrije Universiteit Brussel and Flanders Make, Brussels, Belgium

**Keywords:** transparency, explainability, human-cobot interaction, interactive learning, trust

## Abstract

The number of collaborative robots that perform different tasks in close proximity to humans is increasing. Previous studies showed that enabling non-expert users to program a cobot reduces the cost of robot maintenance and reprogramming. Since this approach is based on an interaction between the cobot and human partners, in this study, we investigate whether making this interaction more transparent can improve the interaction and lead to better performance for non-expert users. To evaluate the proposed methodology, an experiment with 67 participants is conducted. The obtained results show that providing explanation leads to higher performance, in terms of efficiency and efficacy, i.e., the number of times the task is completed without teaching a wrong instruction to the cobot is two times higher when explanations are provided. In addition, providing explanation also increases users’ satisfaction and trust in working with the cobot.

## 1 Introduction

The main method to teach a new task to a cobot is reprogramming, i.e., a programmer writes new code for the cobot to achieve a specific objective. However, this approach is expensive (Bloss, 2016) and needs a lot of tuning so that a slight change in the procedure, e.g., changing the order of objects in the case of an assembly task, can cause the cobot to fail in accomplishing the assembly, if the original program is used, even if the cobot is able to perform all necessary sub-tasks. One solution to both reduce the cost of teaching cobots new tasks and increase their flexibility (being able to perform different but similar tasks) is enabling their human partners to teach them new tasks, such that a cobot learns new high-level tasks from a non-programmer, e.g., an operator in a workshop, who knows the necessary steps to perform the task but does not know how to program the cobot (Billard et al., 2016; Huang and Cakmak, 2017; Stenmark et al., 2017). This way, the costs of cobot maintenance are reduced by transferring behaviors instead of reprogramming them (De Winter et al., 2019). However, this approach requires interactions between cobots and humans, where cobots can clarify, explain, and justify their behaviors for the human partners, and the human partners can transfer their knowledge through feedback to the cobots to help them perform their tasks. Thus, there is a strong need for an efficient mechanism that allows a natural and user-friendly interaction between human partners and cobots. Hence, in this study, a Transparent Graphical User Interface (T-GUI) is designed to provide a bidirectional interaction between a cobot and a human partner such that the cobot can explain why it applies a particular strategy to accomplish a particular task, and the human partner can track the cobot’s actions and provide instructions to it.

One methodology that has previously been used to enable cobots (and other artificial agents and systems) to explain their learned behavior is Explainable Artificial Intelligence (XAI), which tries to enable artificial agents to provide a human understandable explanation that expresses the rationale of the agent and makes it more transparent, so that the agent appears more trustworthy and working with it becomes easier (Adadi and Berrada, 2018; Doran et al., 2017). Thus, one can expect that endowing a cobot with explainability also has the same effect, i.e., enables the cobot to explain and justify its actions, which increases the user’s performance and trust in the cobot.

While XAI is not well defined (Doshi-Velez and Kim, 2017), some researchers tried to define its related notions. For instance, Adadi and Berrada (2018) defined accountability, responsibility, and transparency as XAI’s three main pillars. Doran et al. (2017) also characterized three other notions for it, i.e., opacity, comprehensibility, and interpretability, where interpretability implies model transparency. Interpretability can be influenced by different factors such as model’s transparency and impacts different measurable outcomes, such as trust (Poursabzi-Sangdeh et al., 2018). Thereby, among different notions of XAI, interpretability, and more precisely, transparency, is what we need to enable a cobot to explain its strategy for its human partner. To endow the proposed T-GUI with transparency (as its prime functionality), the knowledge that the cobot acquired during learning how to solve its task (Section 3) is used to produce human-understandable explanations, that explain and justify its actions.

Additionally, since we aimed for bidirectional interactions in which the human partner can also teach new tasks to the cobot, not all necessary assembly instructions are coded into the cobot’s controller, so that it is not able to finish its assembly task due to the lack of required knowledge. Therefore, the proposed T-GUI has another functionality (in addition to explainability) that enables human partners to transfer their knowledge and teach new instructions to the cobot to enable it to accomplish the task that it was not able to perform independently.

To evaluate the efficiency and transparency of the proposed methodology, an experiment is conducted in which the focus was on evaluating users’ ability in teaching correct instructions to the cobot. In addition through provided subjective evaluations, users’ trust in the cobot and their satisfaction with the cobot’s behavior were measured. In this manner, the following questions can be answered: *do the provided explanations through the proposed T-GUI, increase the users*’.

• *performance?*
• *trust in the cobot’s actions?*
• *satisfaction of working with the cobot?*

The remainder of this paper is organized as follows: Section 2 reviews related work. The proposed methodology is explained in Section 3. Section 4 describes the employed experiment and the obtained results. Section 5 discusses the obtained results in more detail and Section 6, finally, concludes this paper.

## 2 Related Work

A variety of models has been proposed in different studies to improve the collaboration between cobots and humans through transparency for different tasks, like cobot teaching and human-cobot collaboration. For example, Chao et al. (2010) proposed a model to enable a robot to learn from a human teacher. Since the author believed the human teacher needs to know in which step the robot is to provide the most informative instructions for it, she proposed a transparent learning process in which the level of the robot’s understanding can be determined in every step. To achieve this, the robot is able to express its uncertainty by related gestures. To evaluate the proposed model, a scenario is used in which users teach names of four symbolic objects to the robot, and later ask the robot whether it remembers these names. The robot is able to answer in five scales, i.e., certain-yes, uncertain-yes, uncertain, uncertain no, or certain-no. To show certain yes or no, it uses nodding and head-shaking, respectively, to show uncertain yes and no, it employs combinations of simultaneous head and body gestures, and for expressing uncertainty it utilizes shrugging. In this way, the human teacher can understand at which level the robot’s understanding is. Although this model enables human teachers to understand whether the robot has learned the task or not, the reason why it did not learn yet is not provided. Additionally, the model does not allow the user to provide support in form of feedback to the robot when it made a mistake, instead the user has to teach the whole task again until the robot finally learns it. In contrast, the proposed model in this study enables the cobot to justify its actions by explaining the reason it is applying them, and the human teacher can directly provide missing instructions to enable the cobot to learn how to continue its task to accomplish it correctly, which speeds up the learning process.

Following the work of Hayes and Scassellati (2016), Roncone et al. (2017) improved the transparent task planner originally proposed by Hayes and Scassellati (2016), which enables both humans and cobots to reason and communicate about the role of each other so that the other agent can act accordingly. To this end, a hierarchical graphical representation of the task is provided to model the task at an abstract level without details of the implementation. In the task’s graphical representation, each sub-task or atomic action, e.g., mounting an object, is shown as a block. While the level of the hierarchy determines the order of sub-tasks/actions, different operators are proposed to show the relation between them, i.e., “→” indicates sequential tasks, “∥” determines parallel tasks, and “⋁” is used to show alternative tasks. During task execution, the cobot can provide feedback to the human partner by highlighting its estimation of the current sub-task, i.e., coloring the related block in the graphical presentation. So that the cognitive load of the human partner, i.e., taking care of roles, is reduced and the task is completed in a shorter time. The main drawback of the study by Roncone et al. (2017) is that the interaction is unidirectional, i.e., the cobot can express its status to the human partner, but the human partner cannot transfer knowledge or missing information to the cobot. In contrast, the model proposed in this paper enables the human partner to add missing instructions to the cobot.

Edmonds et al. (2019) proposed a framework to generate explanations about cobots’ internal decisions. To examine the framework, a scenario is used in which a robot learns from human demonstrations how to open medicine bottles. To this end, three different models are used: 1) a symbolic action planner, which serves as a symbolic representation of the task, 2) a haptic prediction model, which acquires knowledge of the task, e.g., imposed forces and observed human poses, and 3) an improved Earley parsing algorithm which jointly leverages the other two models. In addition, the framework is able to generate explanations. For this, the symbolic action planner provides a mechanistic explanation by visualizing multiple action sequences to describe the process of opening a bottle, i.e., the internal robot gripper state for different actions like approaching the bottle, grasping it, pushing it, etc. The haptic prediction model, on the other hand, provides a functional explanation by visualizing the essential haptic signals to determine the next action. The proposed framework generates explanations in two forms, i.e., a text summary and a visualized description. Based on the obtained results, the latter form is more effective in promoting human trust. Similar to the other mentioned studies, the provided explanations clarify the robot’s actions and status for the human partner, but the employed models do not allow the human partner to interact and transfer knowledge to the robot, i.e., it is a unidirectional interaction, while our model provides a bidirectional interaction through which cobots can provide explanations and human partners can provide instructions.

(Interactive) reinforcement learning is one of the most applied learning models to teach new tasks to cobots, however, in large search spaces, RL-based approaches require a long time to learn. Thus, Senft et al. (2017) proposed a model to make a robot’s learning procedure faster. To this end, he proposed a suggestion and correction system in which a human, as a teacher, provides feedback on a robot’s progress in baking a cake. To bake the cake correctly, the robot should be able to select the correct object in every state, otherwise, the task fails. Since the robot indicates its next action by gazing toward the selected objects or location, the human teacher can estimate its next action, and in case, the robot is going to select an incorrect object, the teacher can intervene by recommending the correct object. For instance, if the robot is gazing at a spoon to indicate that it is going to select the spoon to put it in an oven instead of a bowl containing batter, the teacher can select the correct object through right-clicking on the bowl (in the simulation world) to teach the correct action for this state to the robot. Although the study allowed human partners to transfer their knowledge to the robot, the robot cannot express its decision-making rationale to the human partner, i.e., it lacks transparency.

Previous studies showed that making the cobots’ controllers transparent increases their acceptability, predictability, and trustworthiness. However, to the best of our knowledge, there is no comprehensive model that enables cobots to both reason about their actions and empower human partners to teach cobots new tasks.

## 3 Methodology

**FIGURE 1:**
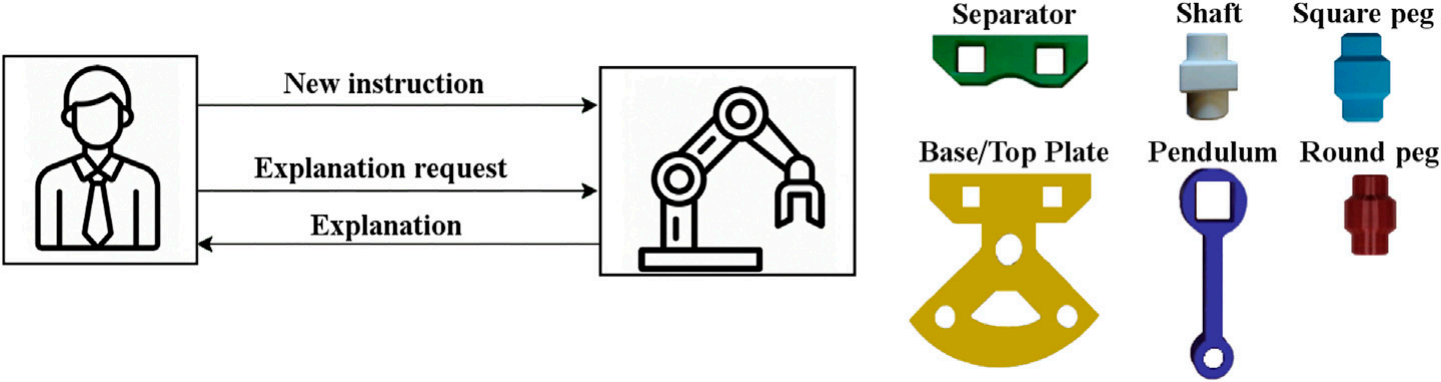
The architecture of the proposed methodology (left) and objects of the Cranfield benchmark (right). The human partner is able to see the cobot’s action and assembly state. Further, the human partner can ask the cobot to explain its actions, which leads to the cobot producing a sentence to explain the reason for the performed action (Section 3.1). In addition, the human partner can directly teach a new instruction to the cobot to enable it to restart the learning procedure with more knowledge about the task, which eventually enables it to complete the task (Section 3.2).

In our previous work (De Winter et al., 2019), we applied an Interactive Reinforcement Learning (IRL) model to enable a cobot to assemble the Cranfield benchmark (Collins et al., 1985). The Cranfield benchmark comprises of 9 objects: base plate, top plate, square peg (×2), round peg (×2), pendulum, separator, and shaft (Figure 1). To assemble the Cranfield benchmark, the cobot needs to follow 16 constraints (Table 1). These constraints come from the fact that when assembling the Cranfield benchmark, the placement of some objects is a prerequisite for the placement of other objects, while for some objects the order of the placement is not important. For instance, the assembly can only start with one of the plates, however, once one of the plates is placed, round pegs, square pegs, and shaft (5 objects) can be placed, while pendulum, separator, and top plate can not be assembled yet. Therefore, in some states of the assembly, several objects can be assembled in parallel, while in some other states, objects should be assembled in a specific order.

**TABLE 1:** The list of required constraints for assembling the Cranfield benchmark.

#	Constraint	#	Constraint
1	Base Plate before Square peg 1	9	Round peg1 before Top Plate
2	Base Plate before Square peg 2	10	Round peg2 before Top Plate
3	Base Plate before Round peg 1	11	Square peg1 before Separator
4	Base Plate before Round peg 2	12	Square peg2 before Separator
5	Base Plate before Shaft	13	Separator before Top Plate
6	Base Plate before Pendulum	14	Shaft before Pendulum
7	Base Plate before Separator	15	Shaft before Top Plate
8	Base Plate before Top Plate	16	Pendulum before Top Plate

The challenge in (De Winter et al., 2019) was to teach the cobot the correct order of the objects to assemble the Cranfield benchmark correctly, which IRL was able to achieve. The applied IRL model generates a Q-table, which shows the appropriateness of selecting each object in different states. This appropriateness value is determined based on the number of violated constraints. Since the applied task is fully constrained, the applied IRL model converges to an assembly sequence that does not violate any constraint. Thus, the cobot can finish the assembly task correctly.

However, if some of the instructions are missing, it is possible that the cobot is not able to completely learn the desired task, in which case it is necessary that the human can provide support, which in turn requires the human to understand the previously executed actions of the cobot. Therefore, we propose a T-GUI which allows the cobot to explain its actions and the human to provide missing instructions to help the cobot finish the task. Both components are explained in more detail in the following subsections.

### 3.1 Cobot’s Explanation for Human Partner

**FIGURE 2:**
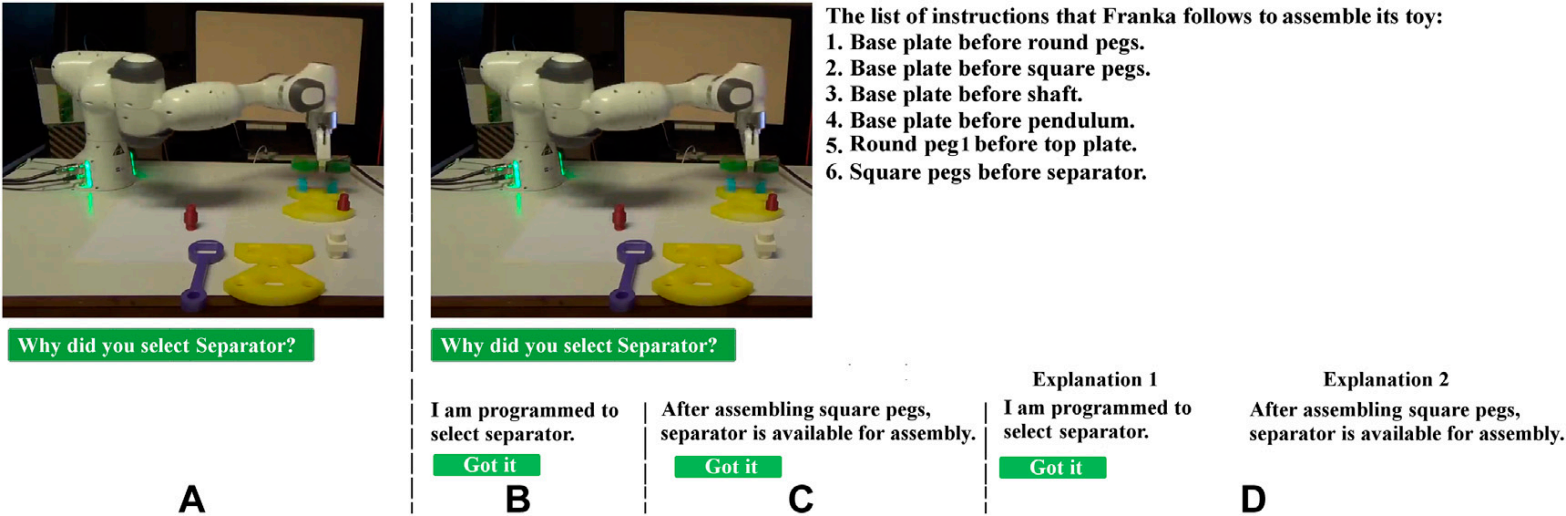
Sub-figure **(A)** shows the default interface, where participants can watch a video showing the cobot is assembling Cranfield benchmark. Meanwhile the button “Why did you select this object?” also is shown, which gives participants the chance to read the cobot’s explanation about its action. By clicking on this button, the video stops, and based on the experiment (between-subjects or within-subjects) one of the other three sub-figures, i.e., **(B–D)**, appears. Sub-figures **(B, C)** are related to the between-subjects design experiment, so that for half of the participants only **(B)** is shown, and for the other half only **(C)** is shown. Sub-figure **(D)** is shown in the within-subjects design experiment, i.e., participants can see explanations generated by the baseline model and the proposed model at the same time. In any sub-figure, by clicking on “Got it” button, the explanation/s disappears, and the video continues (users can again ask the robot for explaining its next actions by clicking on “Why did you select this object?“.

Since there are multiple ways the Cranfield benchmark can be assembled, the human might not immediately understand why the robot is performing a specific action, i.e., picking one object instead of another, when both can theoretically be assembled in parallel, so that it is beneficial, if the robot is able to provide an explanation. To this end, a button which is labeled as “Why did you select this object?” is provided in the proposed T-GUI (Figure 2A) to enable the human partner to ask for an explanation. To answer this question and generate an appropriate explanation, the cobot utilizes previously obtained constraints that indicate in which state which object can be assembled. All generated explanations have the following structure: “After assembling list-of-already-assembled-objects, possible objects for assembly are: list-of-possible-objects-in-this-state”. For instance, if base plate and square pegs are assembled, selecting the separator provides a positive reward, thus if the user asks the cobot why it selected the separator for assembly, the cobot can explain: “After assembling base plate and square pegs, possible objects for assembly are: separator, round peg2, and shaft.” (Figures 2C,D). Thus, by providing this explanation, the human partner can understand which objects are available options for assembly and adjust her expectation, which is the first contribution of this study.

### 3.2 Transferring Human Knowledge to Cobots

**FIGURE 3:**
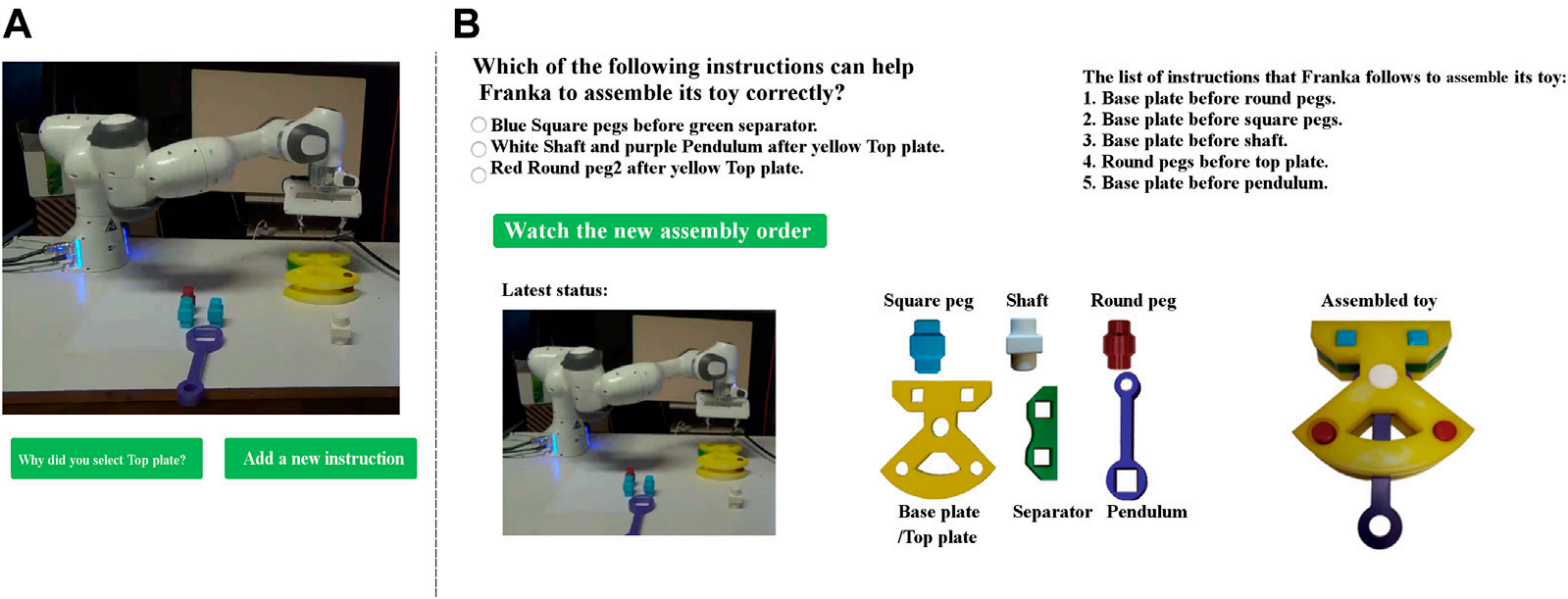
If the cobot stops the assembly task while some objects remain unassembled **(A)**, the user can click on “Add a new instruction” button which leads to a new window **(B)**, where a new instruction can be taught. In the new window **(B)**, the user can see the latest status of the assembly task, the name of different objects, the correctly assembled form of the Cranfield benchmark, and three instructions from which the user can teach one to the cobot (only one instruction is correct). Each instruction provides information about the correct assembly order of two objects, e.g., yellow base Plate before red Round peg. It is worth mentioning that this interface is the same for both B-GUI and T-GUI (Section 4).

The list of constraints that are defined for an assembly task is used as instructions that a cobot can follow to accomplish its task. However, if this list is not fully available before starting the task (due to different reasons, e.g., it is a new task with extra constraints, the human partner does not know all the constraints, or she has forgotten to encode some of them), the cobot can not finish its task correctly, because it is able to select an object that does not violate any of the known constraints but will eventually block the assembly task. In such a situation, if the human partner transfers her knowledge of potential constraints (as new instructions) to the cobot, it can learn to accomplish the task. Thus, the provided T-GUI contains another button, which is labeled as “Add a new instruction” (Figure 3A) through which the user can add a new instruction to the list of the instructions known by the cobot (Figure 3B).

To illustrate such a situation, five constraints of the Cranfield benchmark assembly task are eliminated from the list of instructions initially provided to the cobot, which are: “square peg1 before separator”, “square peg2 before separator”, “shaft before top plate”, “pendulum before top plate”, and “round peg2 before top plate”. In fact, the necessary instructions which have to be considered to start the assembly are given, and the other instructions are chosen by random. In this manner, it is possible that the cobot first places the separator before square pegs (due to lack of constraint “square pegs before separator” (Figure 2A) and then assembles the top plate, which causes some objects to remain unassembled.

Once the cobot stops the assembly, if there are some unassembled objects, the human partner can add a new instruction to help the cobot to assemble the remaining objects via the “Add a new instruction” button (Figure 3A). After clicking on this button, three instructions are shown to the user as possible options to teach to the cobot^1^, however, only one of them is correct. The two incorrect instructions are used to ensure that participants understand the goal of the experiment and aim to help the cobot by selecting the correct instruction. The provided instructions have one of the following structures: “list-of-prerequisite-objects before list-of-possible-objects” or “list-of-possible-objects after list-of-prerequisite-objects”, e.g., “square pegs before separator”, or “separator after square pegs”, respectively.

After the user added a new instruction, the cobot restarts the assembly taking into account the new instruction. Thus, using the provided T-GUI, the human partner is able to teach a new instruction to the cobot without knowing how to program the robot, thereby, enabling the cobot to do a new task or make a previously impossible task feasible, which represents the second contribution of this study.

## 4 Experimental Setup and Obtained Results

To evaluate the efficiency and transparency of the proposed T-GUI an experiment is designed in which a cobot can grasp, transfer, and place all Cranfield benchmark objects individually, and explain the reason for its actions^2^. However, due to several missing constraints (constraints 10, 11, 12, 15, and 16 in Table 1) it can only partially perform the assembly task, i.e., the assembly stops while some objects are still unassembled.

For evaluation purposes, the provided explanation in the T-GUI is compared with a baseline explanation in baseline GUI (B-GUI). B-GUI illustrates a model through which the human partner can transfer knowledge to the cobot, but it is not transparent, i.e., the cobot is not able to explain its strategy to the human partner. Hence, the B-GUI contains the same buttons as the proposed T-GUI except that in response to “Why did you select this object?” it only says “I am programmed to select this object” because it is how cobots usually perform a task, i.e., by following hard-coded instructions.

To explain the experimental procedure for participants, we made a story in which our platform, i.e., Franka^4^ is introduced as a robotic arm that wants to assemble its toy (Cranfield benchmark), but it does not know how to do it, however, participants are able to help it by teaching some instructions to it. In addition, participants are told that they can ask Franka why it selects a specific object in a specific state to understand its strategy.

Due to COVID-19, the experiment could not be run on-site. Thus, an online version of the experiment is provided and participants are recruited through Amazon Mechanical Turk (AMT)^3^ (Crowston, 2012).

As the experiment is conducted online, all possible actions of the cobot in all possible conditions are taped, and based on which instruction the participants teach to the cobot, the related video is shown to them so that they have the feeling that the cobot is performing the taught instruction.

To evaluate the proposed methodology, both objective and subjective evaluations are used. For objective evaluation, the number of participants who were able to add all missing instructions without adding any wrong instructions is checked. For the subjective evaluation, participants are asked to indicate their level of agreement on two scales of explanation satisfaction and trust, recommended for XAI models, from 1 to 5, where 1 indicates “strongly agree” and 5 indicates “strongly disagree”. The applied scales are selected from (Hoffman et al., 2018), which try to measure the participants’ impression about different concepts of a cobot’s behavior and explanation interface, e.g., how much the proposed T-GUI is increasing trust in the cobot and satisfaction with the interaction (Table 2).

**TABLE 2:** Illustration of the applied statements which are selected from (Hoffman et al., 2018) and the comparison of the obtained mean and standard deviation in each item for the proposed and baseline models for the two used experimental designs. Following, Hoffman et al. (2018), 1 indicates strongly agree and 5 indicates strongly disagree, therefore, the lower the rating, the better are the results, except for statement number 14, which is inverse coded.

	#	Statement	Between subjects design				Within subjects design					
			B-GUI		T-GUI		B-GUI		T-GUI		Wilcoxon test	
			M	STD	M	STD	M	STD	M	STD	Z	P
Saticification	1	From the explanation, I understand how the cobot works	1.94	1.02	**1.78**	0.79	1.87	0.94	**1.37**	0.63	−2.44	0.015
	2	This explanation of how the cobot works is satisfying	2.31	1.41	**2.13**	0.81	1.66	0.63	**1.37**	0.48	−2.07	0.038
	3	This explanation of how the cobot works has sufficient detail	2.36	1.3	**2.13**	1.05	2.45	1.02	**1.7**	0.93	−3.31	0.001
	4	This explanation of how the cobot works seems complete	2.47	1.42	**2.3**	1.06	2.12	1.07	**1.75**	0.82	−1.98	0.048
	5	This explanation of how the cobot works tells me how to use it	2.47	1.3	**2.13**	1.04	2.08	1.21	**1.5**	0.91	−2.14	0.032
	6	This explanation of how the cobot works is useful to my goals	2.73	1.28	**2.3**	0.87	2.38	1.06	**1.63**	0.95	−3.35	0.001
	7	This explanation of the cobot shows me how accurate the cobot is	2	1	**1.86**	0.86	2.33	0.96	**1.5**	0.76	−2.96	0.003
	8	This explanation lets me judge when I should trust and not trust the cobot	2.78	1.18	**2.3**	1.14	2.45	0.93	**1.7**	0.97	−3.29	0.001
Trust	9	I am confident in the cobot. I feel that it works well	**2.05**	0.97	2.17	0.88	2.21	0.98	**1.46**	0.64	−3.49	0.000
	10	The outputs (object selection) of the cobot are very predictable	**2.2**	1.18	2.26	0.91	2.37	1.09	**1.83**	0.94	−3.13	0.002
	11	The cobot is very reliable. I can count on it to be correct all the time	**2.47**	1.17	2.65	1.02	2.62	1.17	**2.08**	0.99	−2.67	0.008
	12	I feel safe that when I rely on the cobot I will get the right answers	2.57	1.21	**2.47**	1.2	2.29	0.9	**1.7**	0.78	−2.95	0.003
	13	The cobot is efficient in that it works very quickly	**2.6**	1	2.7	1.2	2.20	0.97	**1.79**	0.86	−2.43	0.015
	14	I am wary of the cobot	**3.05**	1.18	3	1	1.54	0.25	**2.79**	0.22	−2.23	0.026
	15	The cobot can perform the task better than a novice human user	**3.26**	1.32	3.56	1.4	2.54	1.25	**2.2**	1.22	−2.27	0.023
	16	I like using the system for decision making	**2.57**	1.42	2.69	1.36	2.12	1.11	**1.66**	1.06	−2.60	0.009

The bold value in each experimental setting shows that which model performed better in that experimental setting. Thus, in each row, two coloumns are bold, to show the better model in each experimental setting.

The experiment was conducted in two designs that are explained in the following subsections.

### 4.1 Experiment I: Between-Subjects Design

First, a between-subjects design experiment is conducted, where half of the participants interacted with the B-GUI (Figure 2B), and the other half interacted with the proposed T-GUI (Figure 2C). Overall, 71 participants participated in this experiment, however, 29 sessions are discarded due to failure in truth estimation. For instance, we collected the number of times a user had clicked on “Why did you select this object?” and discarded sessions in which the user never clicked on this button, since the applied questionnaires are about the explanations which are only shown after clicking on this button.

We also designed some related questions like “Do you know programming?” and “Did you ever program a cobot?“, to verify if the participants read the questions carefully. Sessions in which users answered “no” and “yes”, respectively, for these questions are discarded. Additionally, we tracked the time each user spent answering the questionnaires, and if this time is not acceptable, we discarded the session. For instance, the average time for answering 16 questions is 95 s for the accepted sessions, while sessions in which users spent less than 75 s are discarded, a double-check also shows that for these very short sessions most of the time only one rate is selected for all of the statements. In the end, 42 sessions are kept, i.e., 21 participants for the baseline and 21 participants for the proposed method. 18 participants are self-reported as female and 24 as male. Their ages ranged between 22 and 46 (*M = 30.77*, *SD = 5.92*), and none of them had related education or occupational background.

#### 4.1.1 Results of Objective Evaluation

The results of the objective evaluation show that in the experiment with B-GUI, nine participants, and in the experiment with the proposed T-GUI, 18 participants selected the correct instructions. Thus, one can conclude, providing explanations clarifies the task and helps participants to better understand the task and thereby give correct instructions.

#### 4.1.2 Results of Subjective Evaluation

To evaluate the proposed GUIs subjectively, we asked users’ impressions through the two scales of explanation satisfaction and trust (Table 2). As obtained results by Shapiro-Wilk test for normality reject the null hypothesis (Table 3), to verify whether participants’ rating to the proposed model and the baseline model are significantly different, Mann-Whiteny U test is applied over participants’ ratings to the models (Table 3), which showed there is no significant difference between B-GUI and T-GUI. However, since the objective results supported the efficiency of the proposed method, we conclude that a lack of comparison may be the reason why the results of the subjective evaluation are insignificant, thus, we repeated the experiment with a within-subjects design to reevaluate the participants’ subjective opinion.

**TABLE 3:** Results of statestical tests in Between-subjects design experiment (*α* = 0.05), which are obtained by summation across all items of each scale.

Scale	Shapiro-Wilk		Mann-Whitney U		
	B-GUI	T-GUI	z-score	*p*-value	U
Explanation Saticification	0.07 (W = 0.90)	0.26 (W = 0.94)	−0.66	0.50	191.5
Trust	0.26 (W = 0.93)	0.07 (W = 0.92)	0.88	0.37	183

### 4.2 Experiment II: Within-Subjects Design

Experiment I is modified to a within-subjects design. To this end, the experimental design is changed so that participants are able to see the explanations for B-GUI and T-GUI in one window (Explanation 1 and Explanation 2 in Figure 2D, respectively). The same truth estimations are used and the experiment is conducted until 25 acceptable sessions are obtained. The self-reported gender is 8 females, 16 males, and one other. Participants’ ages ranged from 18 to 51 (*M* = 36, SD = 7), and none of them had related education or occupational background. To ensure there is no subjects group overlapping, participants are asked if it is the first time they attend the experiment. Additionally, analyzing the demographic data of the participants (age, gender, education, and occupation) there was no overlap between participants in the two experiments. The objective evaluation shows 17 participants are able to select all missing instructions without teaching any wrong instructions to the cobot. It may seem that in the between-subjects experiment more participants gave correct instruction, i.e., 18 out of 21 (in between-subjects design) in comparison to 17 out of 25 (in within-subjects design). However, it is important to note that, participants should give three instructions to the robot to finish the assembly task, and providing a single mistake leads to separating the participant from those that gave correct instructions. In this manner, the overall number of mistakes in the within-subjects design, i.e., 31 mistakes by 25 participants, is comparable with the overall number of mistakes that were made in the between-subjects design, i.e., 29 mistakes by 21 participants.

To evaluate if the obtained results are significant, we applied the Wilcoxon test, since our data suggests a deviation from normality (Table 4, Shapiro-Wilk test). The obtained results by Wilcoxon test for subjective evaluation showed that participants rated the T-GUI significantly higher than the B-GUI for both explanation satisfaction (*z* = − 3.86*,* *p* = 0.000 26) and trust (*z* = − 3.65*,* *p* = 0.000 12) scales as shown in Table 4.

**TABLE 4:** Results of statestical tests in Within-subjects design experiment (*α* = 0.05), which are obtained by summation across all items of each scale.

Scale	Shapiro-Wilk		Wilcoxon	
	B-GUI	T-GUI	z-score	*p*-value
Explanation Saticification	0.51 (W = 0.96)	0.31 (W = 0.95)	−3.86	0.000 12
Trust	0.03 (W = 0.90)	0.03 (W = 0.91)	−3.65	0.000 26

## 5 Discussion

As participants were asked to rate the interaction based on two scales of explanation saticification and trust, obtained results show provided explanations increase users’ trust in the cobot (*z* = − 3.65*,* *p* = 0.00012, Table 4), and also showed participants were satecified by the provided explanations (*z* = − 3.86*,* *p* = 0.000 26, Table 4). To make a deeper look into the obtained results, Wilcoxon test (in within-subjects design experiment) is applied to participants’ ratings to each individual item of the rated scales. Results showed that adding explanation increases users’ understanding of how the cobot works (item 1, *M* = 1.37*,* SD = 0.63*,* *z* = − 2.44*, and* *p* = 0.01) and how to use the cobot (item 5, *M* = 1.5*,* SD = 0.91*,* *z* = − 2.14*, and* *p* = 0.03). In addition, participants believe the provided explanations contain sufficient detail (item 3, *M* = 1.7*,* SD = 0.93*,* *z* = − 3.31*, and* *p* = 0.001), and are complete (item 4, *M* = 1.75*,* SD = 0.82*,* *z* = − 1.98*, and* *p* = 0.04) and satisfying (item 2, *M* = 1.37*,* SD = 0.48*,* *z* = − 2.07*, and* *p* = 0.03). Further, participants believe that based on the provided explanations, it is possible to know when they can trust and when they can not trust the cobot (item 8, *M* = 1.7*,* SD = 0.97*,* *z* = − 3.29*, and* *p* = 0.001), since the explanations show how accurate the cobot is (item 7, *M* = 1.5*,* SD = 0.76*,* *z* = − 2.96*, and* *p* = 0.003).

Moreover, obtained results show participants are confident in the cobot (item 9, *M* = 1.46*,* SD = 0.64*,* *z* = − 3.49*, and* *p* = 0.000) since it works well and quick (item 13, *M* = 1.79*,* SD = 0.86*,* *z* = − 2.43*, and* *p* = 0.01). They also feel safe so that it is possible to rely on the cobot since it gives right answers (item 12, *M* = 1.7*,* SD = 0.78*,* *z* = − 2.95*, and* *p* = 0.003) and does correct actions (based on its knowledge) (item 11, *M* = 2.08*,* SD = 0.99*,* *z* = − 2.67*, and* *p* = 0.008). Further, participants do not wary the cobot (item 14, *M* = 2.79*,* SD = 0.22*,* *z* = − 2.23*, and* *p* = 0.02). Interestingly, they believe they can use the system for decision making (item 16, *M* = 1.66*,* SD = 1.06*,* *z* = − 2.6*, and* *p* = 0.009), which shows how transparent and reliable the provided model is. They also believe the output of the cobot is predictable (item 10, *M* = 1.83*,* SD = 0.94*,* *z* = − 3.13*, and* *p* = 0.002), which might be due to showing the list of the instructions that the cobot follows, i.e., as the proposed T-GUI provides the instructions, participants can read and predict what will be the next assembly object, which also shows how reasonable the cobot’s strategy is.

Thus, one can conclude that transparency, i.e., providing an explanation about cobot’s actions, enhances the performance of the users, leads to more trust in the cobot’s actions, and increases the users’ overall satisfaction with the cobot.

Further, as working with industrial robots is increasing, different programming tools are proposed in the literature, e.g.,Huang and Cakmak (2017) andStenmark et al. (2017) developed models to enable non-expert users to program a robot. Since the obtained results in this study showed that enabling a robot to explain its behaviors improves users’ performance in teaching new tasks to the robot, it is also possible that endowing the proposed models inHuang and Cakmak (2017); Stenmark et al. (2017) with an explanation generation module improves the performance of the non-expert users in teaching new tasks to the robot, which needs to be investigated in future work.

## 6 Conclusion

A transparent interaction between a cobot and a human partner that work on a shared task not only can improve the human’s performance but also make impossible tasks feasible for the cobot. In this study, we show that providing a GUI through which the cobot can explain the reason behind its actions and human partners can transfer their knowledge to the cobot, makes interactions more transparent, and enables the cobot to accomplish a task that it was originally not able to do without the help of an expert programmer. In addition, the results show that the human partner performs better and makes fewer mistakes in teaching, if the cobot explains its behavior. This performance improvement can be due to a better understanding of the task’s detail, which is obtained through the explanations provided by the cobot. Additionally, the obtained results show that providing a transparent GUI increases users’ trust in and satisfaction with the cobot.

As provided explanations in this study are generated based on previously obtained knowledge, it is only possible to generate explanations when the cobot has already learned the task. In future work, we are going to investigate the generation of explanations in an online manner, i.e., while the robot is still trying to learn the task. Additionally, we are planning to allow the human partner to already provide support during the learning utilizing the explanations provided by the cobot. Finally, we will conduct an experiment with a larger population size to increase the representativeness of the results.

## Data Availability

The raw data supporting the conclusion of this article will be made available by the authors, without undue reservation.

## Footnotes

1 To have a uniform input, predefined options are used instead of text input.

2 Details about the employed method and the obtained data are available upon demand.

3 Participants provided their consent before starting the experiment, and IRB approval is not required in our university.

4 Available at https://www.franka.de/technology.
